# Preparation and Mechanism of Alkaline-Activated Coal Gangue-Based Geopolymer Grouting Material

**DOI:** 10.3390/ma19091812

**Published:** 2026-04-29

**Authors:** Keyong Wang, Sihan Guo, Yuying Sun, Kunlin Li, Zhenyue Shi, Qingbiao Wang, Chenglin Tian, Yong Sun

**Affiliations:** 1 School of Resources, Shandong University of Science and Technology, Tai’an 271000, China; skd996607@sdust.edu.cn (K.W.); 202483300037@sdust.edu.cn (S.G.); 202483300055@sdust.edu.cn (Y.S.); skd990748@sdust.edu.cn (Q.W.); skdtcl@126.com (C.T.); skd996768@sdust.edu.cn (Y.S.); 2 Jinan Shansong Engineering Machinery Co., Ltd., Jinan 250101, China; 13573769337@163.com; 3 Laboratory of Rock Burst Prevention Technology and Equipment, Shandong Energy Group Co., Ltd., Jinan 250200, China; 4 Civil Engineering College, Jinan Campus, Shandong Jianzhu University, Jinan 250101, China

**Keywords:** grouting material, coal gangue, alkali excitation, orthogonal test, microscopic analysis

## Abstract

To respond to the national “double carbon” strategic goal, promote the green and low-carbon transformation of the building materials industry, and develop low-carbon and environmentally friendly grouting materials, this study prepared an alkaline-activated coal gangue-based geopolymer grouting material (AACGM). The effects of CG content, alkali activator modulus, and alkali activator content on material fluidity, setting time, compressive strength, and impermeability were systematically studied using orthogonal tests. The optimal mix ratio was determined and the internal mechanism was revealed by microscopic analysis. The results show that the comprehensive performance is the best when the content of CG is 50%, the modulus of alkali activator is 1.6, and the content of alkali activator is 14%. The primary and secondary order of influence of various factors on the performance is as follows: CG content > alkali activator content > alkali activator modulus. Microscopic analysis revealed that the hydrolysis polymerization products of the material are mainly C-S-H, C-(N)-A-S-H gel, and zeolite-like phase, forming a dense three-dimensional network structure, which is the internal mechanism of its good mechanical and impermeability properties. This study provides a new concept for the utilization of CG, and the prepared materials are of great significance in the field of grouting reinforcement in underground engineering.

## 1. Introduction

With the increasing energy and environmental crisis and continuous improvement of environmental protection requirements, the demand for grouting projects continues to grow. The green transformation of traditional high-energy-consumption and high-emission industries has become an inevitable trend. Traditional grouting materials are primarily cement-based [1], with the advantages of stable strength and convenient construction. However, there are drawbacks such as high raw material consumption, high carbon emissions in the production process, insufficient flexibility in setting time adjustment, and poor corrosion resistance [2,3], which make it difficult to meet the needs of low-carbon environmental protection and high-performance materials for engineering construction. Therefore, there is an urgent need to develop new grouting materials with low carbon content for environmental protection. At the same time, in China, as a large coal-producing country, the coal gangue (CG) produced in the process of coal mining and washing has become one of the largest and most concentrated industrial solid wastes, with a cumulative stock of 7 billion tons [4,5]. The long-term storage of large amounts of CG not only occupies valuable land resources but also causes safety hazards and pollutes the environment [6,7,8]. Therefore, the use of CG to prepare geopolymer grouting materials [9,10,11] can not only effectively alleviate the environmental pressure caused by its storage but also provide a new type of green grouting material for engineering construction.

To date, domestic and foreign scholars have made some progress in the preparation and performance of alkali-activated CG materials. Studies have shown that proper physical activation, chemical activation, or treatment can effectively improve the activity of CG [12,13,14,15], so that it undergoes a depolymerization-condensation reaction in an alkaline environment to form an inorganic polymer with a three-dimensional network structure. Hongqiang [16] and Ling [17] confirmed that CG has cementitious potential under alkali excitation conditions and improved the basic mechanical properties of the material by optimizing the mix ratio. However, existing research is limited and focuses on a single-factor test. Under the synergistic effects of multiple factors, the comprehensive influence of various factors on the workability, mechanical strength, and durability of grouting materials remains unclear. Moreover, the internal correlation mechanism between the macroscopic properties of materials and the evolution of microscopic gel structure still lacks a systematic explanation.

In this study, CG, slag, and fly ash (FA) were used as the primary raw materials. Through an orthogonal test, the influence of three key factors—CG content, alkali activator modulus, and alkali activator content—on the fluidity, setting time, compressive strength, and impermeability of grouting materials was systematically explored. Compared with existing research, this study systematically evaluated the interaction of these three factors on the comprehensive performance of grouting materials for the first time, and the orthogonal matrix analysis method was used to determine the optimal mix ratio for comprehensive performance. Combined with microscopic analysis, the internal relationship between the gel structure and macroscopic properties was revealed, and the formation mechanism of the gel structure was clarified. This can provide a theoretical basis and technical support for the preparation and application of high-performance CG-based geopolymer grouting materials.

## 2. Materials and Methods

### 2.1. Raw Materials

**Figure 1:**
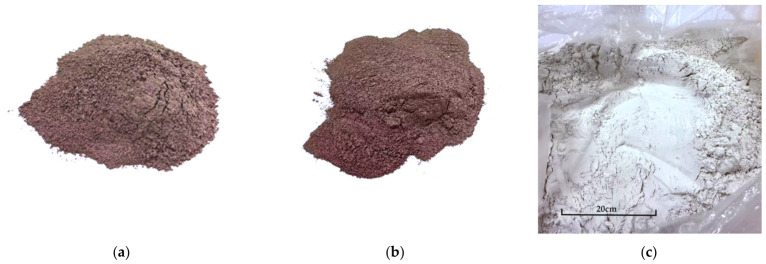
(**a**) CG powder; (**b**) Grade II FA; (**c**) S75 slag. The scale represents approximately 20 cm, indicating the size of the slag heap.

The CG from Zhengzhou, Henan Province, was selected for the test, and a jaw crusher, hammer crusher, and mill were used for treatment. A CG powder that met the requirements was obtained by 300-mesh square hole screening for subsequent application. Grade II FA was selected as the FA. S75 grade slag was selected as the slag. The material samples are shown in Figure 1. The chemical compositions of the materials are listed in Table 1.

The chemical composition of the raw materials shown in Table 1 was determined by using an X-ray fluorescence spectrometer (ZSX Primus III NEXT, XRF) produced by Tokyo Rigaku, Japan.

The alkali activator was a sodium silicate solution, and commercially available waterglass was used in this experiment. The modulus of the alkali activator was adjusted using sodium hydroxide. After sufficient mixing, the adjusted alkali activator solution was allowed to stand for 24 h to eliminate bubbles and allow a full reaction.

### 2.2. Test Mix Proportions and Specimen Preparation

#### 2.2.1. Preliminary Test

In the pre-test, it was found that when the liquid–solid ratio was 0.4, the CG-based geopolymer was too viscous to mix and pour after stirring. When it was greater than 0.5, the fluidity of the CG-based geopolymer was excessively high, and the mechanical strength was significantly reduced owing to the increase in porosity. Therefore, to maintain a controllable research range in the orthogonal test and focus on the three main factors of CG content, alkali activator modulus and alkali activator content, the liquid–solid ratio in this study was fixed at 0.45. This ensures that all the ratios in the orthogonal test have sufficient work required for practical applications so that the influence of the target variable can be studied more independently.

When the content of CG is 10% and 20%, the excitation effect of the CG-based geopolymer is not good, and it cannot be formed after 24 h, but it can achieve a good forming effect at 30%. When the modulus of the alkali activator is 1.0, the CG-based geopolymer exhibits instantaneous condensation and cannot continue to solidify. When the modulus of the alkali activator is greater than 2.0, the strength of the CG-based geopolymer is low.

#### 2.2.2. Orthogonal Test

The effects of CG content, alkali activator modulus, and alkali activator content on the fluidity, setting time, compressive strength, and impermeability of CG-based geopolymer grouting materials were studied. Three different levels of experimental conditions were used for the orthogonal tests.

CG, slag, and FA were used as solid waste silicon-aluminum materials to prepare geopolymer grouting materials. The sum of CG and slag contents in solid waste silicon-aluminum materials was fixed at 90%, and the FA content was 10%. Different CG content (30%, 50%, 70%), alkali activator modulus (1.2, 1.6, 2.0), and alkali activator content (12%, 14%, 16%) were used to prepare specimens. The effects of various factors on the fluidity, setting time, compressive strength, and impermeability of the material were determined by an orthogonal test. The optimal factor combination level for improving the overall performance of the material was determined (Table 2).

**Figure 2:**
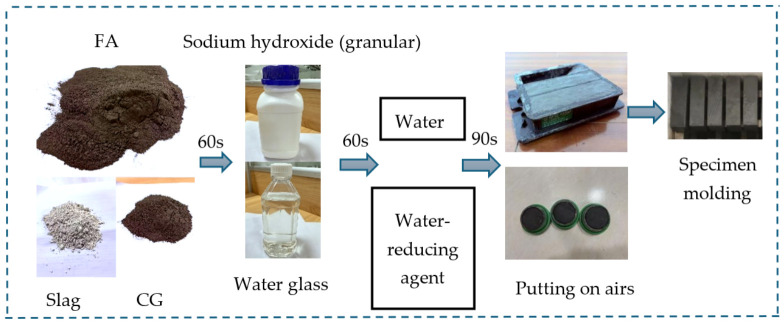
Test specimen preparation.

According to the calculated mix ratio, the CG, slag powder, FA, alkali activator, sodium oxide, water, and water-reducing agent are weighed and the cement paste mixer (NJ-160A) produced by Wuxi Construction Test Equipment Co., Ltd., Wuxi, Jiangsu Province, China was used for mixing. A small vibration was applied by a vibration table for 30 s, discharging the internal air of the specimen to enhance its compactness and structural strength. Then, a plastic film was used for sealing to effectively prevent water loss and ensure the maintenance quality of the specimen. The surface of the specimen was covered after vibrating and compacting. After being placed in a cool area for 24 h, the specimen was demoulded and numbered. The prepared specimen was placed in a standard curing box for curing, and the temperature was controlled at approximately 20 ± 2 °C. The preparation process of the specimen is shown in Figure 2.

The range of test parameters was determined based on a previous exploratory test and the literature research, which ensured that the commonly used interval of the project was covered. To ensure the reliability and repeatability of the test results, three tests were performed for each mix ratio specified in Table 3. The values in Table 4 represent the arithmetic mean values of these three independent measurements, and the error is controlled within 5 %, which meets the requirements.

### 2.3. Test Method

#### 2.3.1. Flow Test

The thoroughly mixed mortar was poured into a truncated cone mold, the surface was leveled, and the mold was lifted vertically. After the mortar flowed freely on the glass plate for 30 s, the maximum spread diameter was measured in mutually perpendicular directions. The average of two measurements was taken as the final result. The flow test was conducted and evaluated strictly in accordance with the relevant provisions of the national standard GB/T 8077-2023 “Test Methods for Homogeneity of Concrete Admixtures” [18].

#### 2.3.2. Setting Time Test

The cement paste was prepared according to the standard consistency water requirement. After filling the mold and leveling the surface, the mold was immediately placed in a humidified curing chamber. The time when all the cement was added to water was recorded as the start of the setting time. When the test needle sank to 4 ± 1 mm from the base plate, the cement reached its initial setting; when the test needle sank 0.5 mm into the specimen, the final setting was reached. These times were recorded as the initial and final setting times, respectively. Setting time testing strictly adhered to the relevant provisions of the national standard GB/T 1346-2024 “Standard Test Methods for Standard Consistency Water Requirement, Setting Time, and Stability of Cement” [19], with results evaluated accordingly.

#### 2.3.3. Compressive Strength Test

Compressive strength tests were conducted on cured specimens measuring 40 mm × 40 mm × 160 mm, and the average value for each group of specimens was recorded. The compressive strength of specimens was strictly evaluated in accordance with the relevant provisions of the national standard GB/T 17671-2021 “Test Methods for Strength of Cement Mortar (ISO Method)” [20].

#### 2.3.4. Water Permeability Test

After curing, the specimens were removed and their surfaces were allowed to dry. The ends were sealed with a sealing material, and they were placed in a mortar permeability tester for water permeability testing. Pressure was applied, starting at 0.2 MPa, maintained for 2 h, and then increased to 0.3 MPa. Subsequently, the pressure was increased by 0.1 MPa every hour. When water seepage appeared on the end faces of three of the six specimens, the test was immediately terminated and the current water pressure was recorded. If water leakage was observed around the specimen periphery during testing, the test was halted and the specimen resealed. Permeability performance was evaluated strictly in accordance with the testing procedures and results assessment outlined in JGJ/T70-2009 “Standard Test Methods for Basic Properties of Building Mortars” [21].

**Figure 3:**
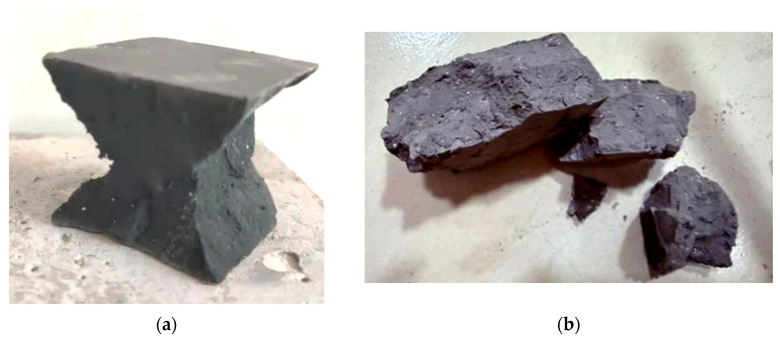
Failure modes: (**a**) failure mode of compression test; (**b**) Failure Modes of Impermeability Test.

The failure modes of the specimens after compression test and impermeability test are shown in Figure 3.

#### 2.3.5. Physicochemical Investigations

The test was performed using an X-ray diffractometer (D8 DISCOVER, XRD) produced by Brook, Karlsruhe, Germany. A Cu target was used for excitation, Kα radiation was used as the ray source, the scanning speed was set to 5°/min, and the scanning range of the diffraction angle covered 5° to 70°. Infrared spectroscopy was performed using a Fourier transform infrared spectrometer (Lyza 7000, FTIR) produced by Graz Anton Paar, Austria. The wavenumber range of spectral acquisition was set to 400 cm^−1^ to 4000 cm^−1^, and the resolution was 4 cm^−1^. Sample preparation was performed using the KBr particle method. The microstructures of the samples with the optimal mix ratio were observed using a scanning electron microscope (Apreo 2S, SEM) produced by Seymour Fisher Technology in Waltham, MA, USA, and the acceleration voltage was 5.0 kV. Gold spray treatment was performed before observation. The microstructures of the 9 samples were observed using a SEM (Sigma 360) produced by Zeiss, Oberkohen, Germany, and the acceleration voltage was 3.0 kV. Gold spray treatment was performed before observation.

## 3. Results

The CG content, modulus of alkali activator, and content of alkali activator were taken as the main test factors, and the four indexes of fluidity, setting time, compressive strength, and impermeability were taken as the important indexes to measure the test. The effect of each test factor on the index was determined based on the test results. The data from the orthogonal test results were analyzed using range analysis [22,23] and orthogonal matrix analysis [24].

### 3.1. Results of Orthogonal Test

The orthogonal test results based on the three factors and three different levels of experimental conditions are presented in Table 4.

A range analysis was performed based on the test results, as shown in Table 5.

#### 3.1.1. Analysis of Fluidity Test Results

According to the results in Table 5, the primary and secondary factors affecting the fluidity of the slurry were as follows: alkali activator modulus > CG content > alkali activator content, where the alkali activator modulus showed the greatest influence on the fluidity of the slurry. The fluidity of the slurry was the largest when the CG content was 30%, the alkali activator modulus was 2.0, and the alkali activator content was 16%.

**Figure 4:**
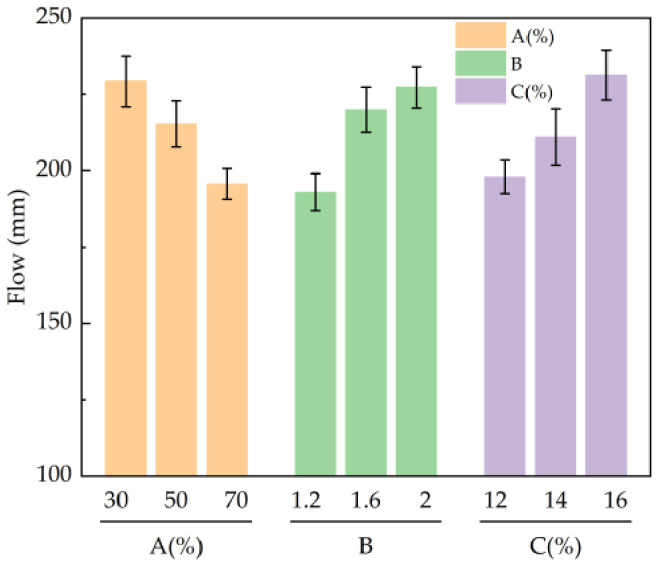
Influence of various factors on slurry fluidity: A: CG admixture ratio; B: alkali activator modulus; C: Alkali Activator Dosage.

It can be seen from Figure 4 that, as the content of CG increased from 30% to 70%, the fluidity of slurry decreased from 229.33 mm to 195.67 mm. The fluidity of the slurry was the largest when the CG content was 30%. As the modulus of the alkali activator increased from 1.2 to 2.0, the fluidity of the slurry increased from 193 mm to 227.33 mm. An increase in the modulus of the alkali activator increased the concentration of silicate, enhanced the electrostatic repulsion between particles, and improved fluidity. The fluidity of the slurry increased from 198 mm to 231.33 mm when the content of the alkali activator increased from 12% to 16%. This is attributed to the increase in silicate ions caused by the increase in dosage, which reduced the yield stress and thixotropic characteristics of the slurry [25]. Increases in the alkali activator modulus and alkali activator content are beneficial for fluidity; however, the influence of the modulus is relatively low and limited.

#### 3.1.2. Analysis of Condensation Time Results

According to the results in Table 5, the influence of various factors on the setting time of the slurry was as follows: CG content > alkali activator content > alkali activator modulus, where CG content had the greatest influence on the setting time of the slurry. The setting time of the slurry was the shortest when the dosage of CG was 30%, the modulus of alkali activator was 1.2, and the dosage of alkali activator was 16%.

**Figure 5:**
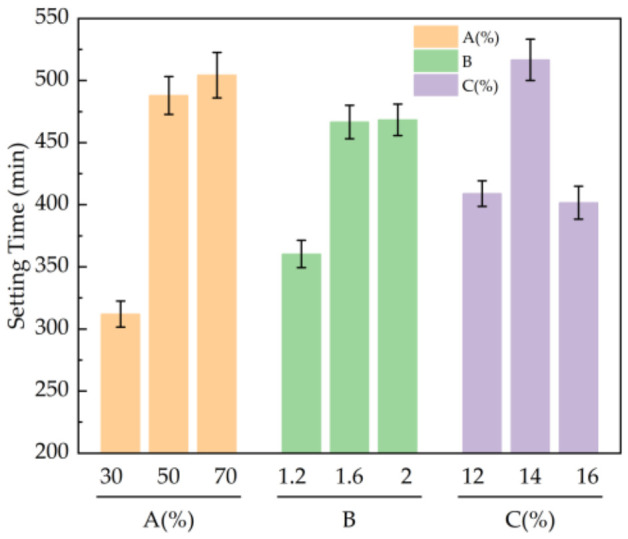
Influence of various factors on the setting time of slurry: A: CG admixture ratio; B: alkali activator modulus; C: Alkali Activator Dosage.

It can be seen from Figure 5 that as the CG content increased from 30% to 70%, the setting time of the slurry increased due to the fact that the active silicon-aluminum component in the CG decreased with an increase in the content, and the activity in the early stage of the reaction was insufficient. With an increase in the modulus of the alkali activator from 1.2 to 2.0, the setting time of the slurry increased because of the increase in the proportion of silicon oxygen tetrahedron polymer in the high-modulus alkali activator, which inhibited the effective reaction between silicate ions and slag hydrolysis products. As the amount of alkali activator increased from 12% to 16%, the setting time of the slurry increased first and then decreased. When the amount of alkali activator was 14%, the setting time of the slurry was 516.67 min. Compared to the case of 16% alkali activator, the setting time increased by 27%. The setting time is highly sensitive to changes in the activator content, mainly because the change in alkalinity affects the dissolution rate of aluminosilicate. In engineering applications, the fluctuation range of activator content can be controlled within ±1%, and fine-tuning can be combined with a retarder or accelerator to ensure the stability of construction.

#### 3.1.3. Analysis of Compressive Strength Test Results

According to the results in Table 5, the primary and secondary influences of various factors on the compressive strength of the stone body were as follows: CG content > alkali activator modulus > alkali activator content, where CG content had the greatest influence on the compressive strength of the stone body. The compressive strength of the stone body was the largest when the CG content was 30%, the alkali activator modulus was 1.2, and the alkali activator content was 14%.

**Figure 6:**
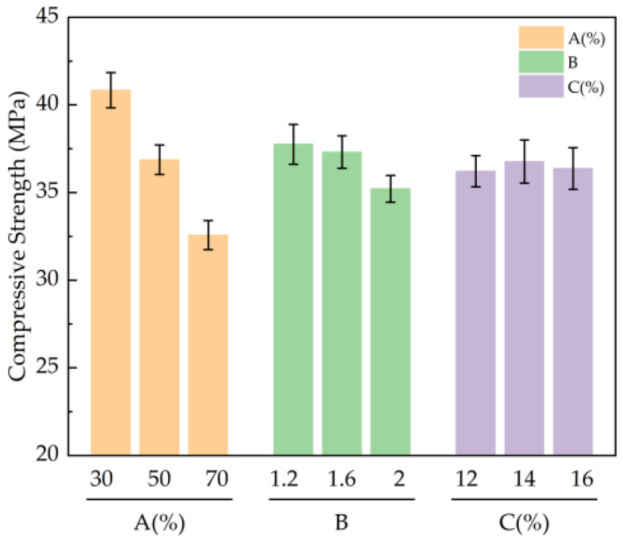
Influence of various factors on the compressive strength of the stone body: A: CG admixture ratio; B: alkali activator modulus; C: Alkali Activator Dosage.

From Figure 6, it can be seen that with an increase in CG content from 30% to 70%, the compressive strength decreased from 40.84 MPa to 32.57 MPa. Owing to the low activity of CG and FA, the active silicon and aluminum substances generated by early hydrolysis were relatively small, resulting in a decrease in the early strength with an increase in dosage, but the later strength gradually increased with curing time. With an increase in the modulus of the alkali activator, the compressive strength of the stone body decreased gradually. When the modulus of the alkali activator reached 1.2, the compressive strength of the stone body was the highest at 37.75 MPa. When the alkali activator content was 12%, the compressive strength of the stone body was the lowest at 36.21 MPa. When the content increased from 12% to 14%, the compressive strength increased by 1.5%. When it continued to increase to 16%, the strength decreased by 1%. Adding an appropriate amount of alkali activator can promote the formation of C-S-H, C-A-S-H, and other gels in the system, which is conducive to improving the early strength.

Because there is no uniform strength standard for such materials at home or abroad, their performance requirements must be determined according to specific engineering scenarios. For a general coal mine roadway surrounding rock grouting reinforcement project, the 28 d compressive strength of the material must reach 20–30 MPa to meet the basic needs of the surrounding rock bearing capacity [26]. The results of this study show that even if the CG content is as high as 70%, its 28 d compressive strength can still reach 32.57 MPa, which significantly exceeds the above engineering foundation threshold. Therefore, in practical engineering, as long as the strength safety threshold of the foundation is satisfied, it is feasible to appropriately increase the amount of CG to improve the utilization rate of solid waste.

#### 3.1.4. Analysis of Impermeability Test Results

According to the results in Table 5, the primary and secondary influences of various factors on the impermeability of the stone body were as follows: CG content > alkali activator modulus > alkali activator content, where CG content had the greatest influence on the impermeability of the stone body. The impermeability of the stone body was best when the content of CG was 30%, the modulus of alkali activator was 1.2, and the content of alkali activator was 14%.

**Figure 7:**
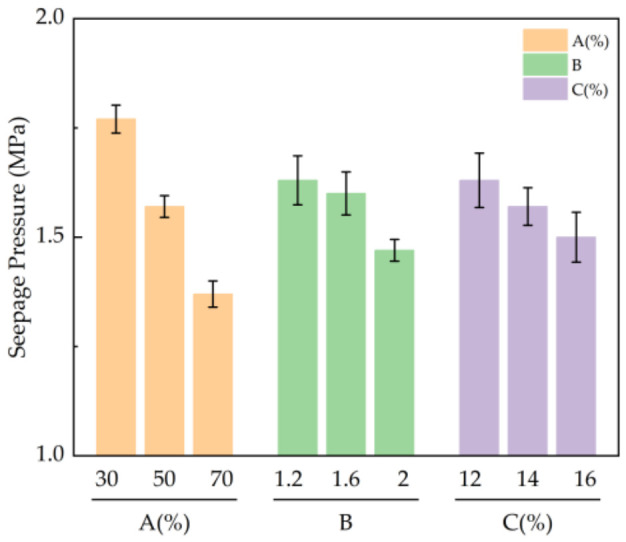
Influence of various factors on the permeable pressure of the stone body: A: CG admixture ratio; B: alkali activator modulus; C: Alkali Activator Dosage.

From Figure 7, it can be seen that as the content increased from 30% to 70%, the permeability pressure decreased by 22.6%, mainly because of the reduction in active substances caused by the reduction in slag, which made it difficult to form a dense structure, and the impermeability decreased. When the modulus of alkali activator reached 1.2, the water permeability pressure of the stone body was the highest at 1.63 MPa, which is 7.4% higher than that of the test group with a modulus of alkali activator of 2.0. When the modulus is high, most of the hydrolysis products participate in the polymerization reaction, and the remaining polymer silicon oxygen tetrahedron makes a limited contribution to the impermeability in the later stage of the reaction. As the amount of alkali activator increased from 12% to 16%, the water permeability pressure decreased by 8%. It can be concluded that a lower amount of water glass is helpful in improving impermeability. Excessive alkali activator interferes with the hydrolysis of slag, produces free alkali, and affects the polymerization reaction and structural compactness.

### 3.2. Analysis of Optimal Mix Ratio of Geopolymer Grouting Material

#### 3.2.1. Orthogonal Matrix Analysis Method

In the range analysis, if it only relies on a single index to determine the optimal mix ratio, it often leads to one-sided results. Therefore, the orthogonal matrix analysis method is used to determine the optimal combination scheme by calculating the weight of each factor level to the target index. The specific steps are as follows:

Test investigation index matrix: If there are m factors in the orthogonal test, each factor has *n* levels, and the average value of the test index at the *j*th level of the factor $A_{i}$ is $k_{ij}$; if the test index of the test result is the larger the better, then $K_{ij}{=k}_{ij}$, otherwise, $K_{ij}=1/k_{ij}$, and establish the matrix of Equation (1).

The factor layer matrix: Let $T_{ij}=1/\sum_{i=1}^{n}K_{ij}$, and establish the matrix of Equation (1).

The horizontal layer matrix: the range of the factor $A_{i}$ in the orthogonal experiment is $s_{i}$, let $S_{i}=s_{i}/\sum_{i=1}^{m}s_{i}$, and establish the matrix of Equation (1).

(1) $$M=\left[\begin{array}{cc} k_{11}\;\;\;\;\;0\;\;\;\;\;0\;\;\;\;\;\ldots \; & \;\;0\\ k_{12}\;\;\;\;\;0\;\;\;\;\;0\;\;\;\;\;\ldots & \;\;0\\ \ldots \;\;\;\;\;\ldots \;\;\;\;\;\ldots \;\;\;\;\;\ldots & \;\;\ldots \\ k_{1n}\;\;\;\;\;0\;\;\;\;\;\;0\;\;\;\;\;\ldots & \;\;0\\ 0\;\;\;\;\;\;k_{21}\;\;\;\;\;0\;\;\;\;\;\ldots & \;\;0\\ 0\;\;\;\;\;\;k_{22}\;\;\;\;\;0\;\;\;\;\;\ldots & \;\;0\\ \ldots \;\;\;\;\ldots \;\;\;\;\;\ldots \;\;\;\;\;\ldots & \ldots \\ 0\;\;\;\;\;\;k_{2n}\;\;\;\;\;0\;\;\;\;\;\ldots & 0\\ \ldots \;\;\;\;\ldots \;\;\;\;\;\ldots \;\;\;\;\;\ldots & \ldots \\ 0\;\;\;\;\;\;\;\;0\;\;\;\;\;\;\;0\;\;\;\;\;\;\ldots & k_{m1}\\ 0\;\;\;\;\;\;\;\;0\;\;\;\;\;\;\;0\;\;\;\;\;\;\ldots & k_{m2}\\ \ldots \;\;\;\;\;\ldots \;\;\;\;\;\ldots \;\;\;\;\;\ldots & \ldots \\ 0\;\;\;\;\;\;\;\;\;0\;\;\;\;\;\;\;0\;\;\;\;\;\;\ldots & k_{mn} \end{array}\right]\;T=\left[\begin{array}{cc} T_{1}\;\;\;\;0\;\;\;\;\;\ldots & 0\\ 0\;\;\;\;{\;T}_{2}\;\;\;\;\ldots & 0\\ \ldots \;\;\;\;\ldots \;\;\;\;\ldots \; & \ldots \\ 0\;\;\;\;\;\;0\;\;\;\;\;\ldots & T_{m} \end{array}\right]\;S=\left[\begin{array}{c} S_{1}\\ S_{2}\\ \ldots \\ S_{m} \end{array}\right]$$

The weight matrix affecting the test index is defined as: K=MTS

(2) $$\;k^{T}=(k_{1},k_{2},\ldots ,k_{n})$$

In the matrix listed above, $k_{1}=K_{11}T_{1}S_{1}$, $K_{11}T_{1}$ is $K_{11}/\sum_{i=1}^{n}K_{ij}$, which is the ratio of the index value of the first level of factor A1 to the sum of the index values of all levels of factor $A_{1}$; $S_{1}$ is the ratio of the range of factor $A_{1}$ to the sum of the range of all factors. The product of the two comprehensively reflects the degree of influence of the level on the target index and the range of the factor. By calculating the weight of each level, the optimal scheme can be determined by sorting, and the primary and secondary relationships of factors can be clarified.

#### 3.2.2. Analysis of Effect

According to the orthogonal matrix analysis method (1) and (2), the data from Table 5 were processed, and the weight matrices *k*_1_, *k*_2_, *k*_3_, and *k*_4_ of each influencing factor were obtained, as follows:

(3) $$k_{1}=\left[\begin{array}{c} 0.11885\\ 0.11159\\ 0.10141\\ 0.10201\\ 0.11628\\ 0.12016\\ 0.10161\\ 0.10828\\ 0.11871 \end{array}\right]=\left[\begin{array}{c} A_{1}\\ A_{2}\\ A_{3}\\ B_{1}\\ B_{2}\\ B_{3}\\ C_{1}\\ C_{2}\\ C_{3} \end{array}\right]k_{2}=\left[\begin{array}{c} 0.11125\\ 0.17400\\ 0.17983\\ 0.07215\\ 0.09344\\ 0.09377\\ 0.08720\\ 0.11016\\ 0.08564 \end{array}\right]=\left[\begin{array}{c} A_{1}\\ A_{2}\\ A_{3}\\ B_{1}\\ B_{2}\\ B_{3}\\ C_{1}\\ C_{2}\\ C_{3} \end{array}\right]\;\;k_{3}=\left[\begin{array}{c} 0.26913\\ 0.24297\\ 0.21463\\ 0.07640\\ 0.07551\\ 0.07126\\ 0.01630\\ 0.01655\\ 0.01637 \end{array}\right]=\left[\begin{array}{c} A_{1}\\ A_{2}\\ A_{3}\\ B_{1}\\ B_{2}\\ B_{3}\\ C_{1}\\ C_{2}\\ C_{3} \end{array}\right]k_{4}=\left[\begin{array}{c} 0.21785\\ 0.19323\\ 0.16862\\ 0.08042\\ 0.07894\\ 0.07253\\ 0.06534\\ 0.06294\\ 0.06013 \end{array}\right]=\left[\begin{array}{c} A_{1}\\ A_{2}\\ A_{3}\\ B_{1}\\ B_{2}\\ B_{3}\\ C_{1}\\ C_{2}\\ C_{3} \end{array}\right]$$

According to the weight matrix *k*_1_, the slurry fluidity was the largest when the CG content was 30%, the alkali activator modulus was 2.0, and the alkali activator content was 16%.

Similarly, according to the weight matrix *k*_2_, the setting time of slurry was the shortest when the dosage of CG was 30%, the modulus of alkali activator was 1.2, and the dosage of alkali activator was 16%.

According to the weight matrix *k*_3_, the compressive strength of the stone body was the largest when the CG content was 30%, the alkali activator modulus was 1.2, and the alkali activator content was 14%.

According to the weight matrix *k*_4_, the impermeability of the stone body was the best when the content of CG was 30%, the modulus of alkali activator was 1.2, and the content of alkali activator was 12%. This conclusion is consistent with the range analysis.

The optimal performance of the geopolymer was determined by a multi-index comprehensive evaluation; thus, the total weight matrix *k_a_* covering the four evaluation indexes was constructed according to Equation (4). It can be seen from *k_a_* that the optimal scheme is as follows: CG content 50%, alkali activator modules 1.6, alkali activator content 14%, where the order of influence of each factor is CG content > alkali activator content > alkali activator modulus. The proportions of the influence of each factor were 52.54%, 26.28%, and 21.18%, respectively.

(4) $${k_{a}=(k_{1}+k_{2}+k}_{3}+k_{4})/4=\left[\begin{array}{c} 0.17927\\ 0.18045\\ 0.16612\\ 0.08275\\ 0.09104\\ 0.08943\\ 0.06761\\ 0.07448\\ 0.07021 \end{array}\right]=\left[\begin{array}{c} A_{1}\\ A_{2}\\ A_{3}\\ B_{1}\\ B_{2}\\ B_{3}\\ C_{1}\\ C_{2}\\ C_{3} \end{array}\right]$$

The fluidity, setting time, compressive strength, and impermeability of the ZJ specimen prepared according to the optimal scheme were measured and are listed in Table 6.

### 3.3. Physicochemical Results of Geopolymer Grouting Materials

According to the optimal mix ratio, the experimental group was set up to analyze the results of physical and chemical analyses.

#### 3.3.1. XRD Results

**Figure 8:**
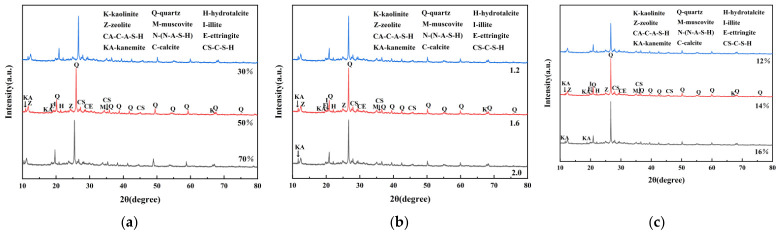
XRD spectra: (**a**) different dosages of CG; (**b**) different alkali activator moduli; (**c**) different alkali activator content.

To clarify the mineral composition and hydrolysis polymerization products in CG and geopolymer grouting materials and to explore the influence of different factors on the mineral, gel, and crystal phase structures of geopolymer grouting materials, the geopolymer grouting material was analyzed using XRD. Figure 8 shows the 28 d XRD pattern of the AACGM.

In Figure 8, a broad dispersion peak can be observed in the range of 2θ = 20~40° in the spectrum, which indicates the presence of amorphous phase materials such as C-S-H and C-(N)-A-S-H [27,28]. In addition, characteristic peaks with low intensity can also be seen at 2θ = 12°, 21°, 27°, 35° and 49°. These peaks also indicate the formation of new crystalline and amorphous phases.

Figure 8a shows that the addition of CG causes the diffraction peak to shift to the left [29], indicating that the substitution of heteroatoms leads to the expansion of the kaolinite lattice. With an increase in CG content from 30% to 70%, the characteristic peak of quartz increased, and the area of the dispersion peak decreased. Combined with Table 5, when the content of CG was 30%, the strength was the highest at 40.84 MPa, and the corresponding dispersion peak area was the largest; when the content was 70%, the strength decreased to 32.57 MPa, and the dispersion peak area decreased significantly. This indicates that excessive CG content will dilute the active component, reduce the total amount of hydration gel, and lead to a decrease in strength. The characteristic peaks of mica and illite in the XRD pattern still exist, indicating that they have good stability and no negative impact on the stone body. Figure 8b shows that when the modulus increased from 1.2 to 2.0, the C-S-H diffraction peak near 27° gradually sharpened, and the degree of order of the gel increased. However, as shown in Table 5, the highest strength was 37.75 MPa when the modulus was 1.2, and the lowest strength was 35.21 MPa when the modulus was 2.0. This indicates that an excessively high order may destroy the continuity of the gel network, and excessive local crystallization degrades strength. Figure 8c and Table 5 show that when the content increased from 12% to 14%, the C-S-H diffraction peak sharpened, the order improved, and the strength increased from 36.21 MPa to 36.77 MPa. When the content increased to 16%, the diffraction peak further sharpened, but the strength fell back to 36.37 MPa, and a weak calcium carbonate peak appeared. This indicates that excessive alkali will interfere with the hydrolysis of slag, destroy the continuity of gel, and generate carbonate to consume alkalinity. In this experiment, an alkali content of 14% was the best.

#### 3.3.2. FTIR Results

**Figure 9:**
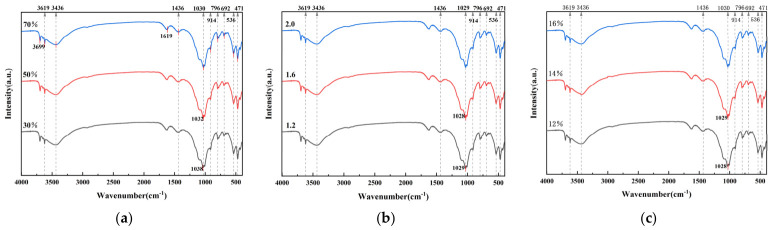
FTIR spectra: (**a**) different dosages of CG; (**b**) modulus of different alkali activators; (**c**) different alkali activator content.

The mineral phase structure characteristics of the original CG and geopolymer grouting material system and evolution of key functional groups in the hydrolysis polymerization reaction process were systematically analyzed using a Fourier infrared spectrometer. Through the synergistic application of XRD analysis, the relationship between various influencing factors and the formation mechanism of hydrolytic polymerization products in geopolymer grouting materials was further revealed. Figure 9 shows the FTIR spectra of AACGM in the wavenumber range of 400–4000 cm^−1^.

Figure 9a shows that with an increase in slag content, the absorption peak of Si-O-T asymmetric stretching vibration at approximately 1030 cm^−1^ shifts to a lower wavenumber. Its highly active calcium-silicon component promotes the dissolution and recombination of active silicon-aluminum substances and accelerates the formation of Si-O-T structure and C-(N)-A-S-H gel. The stretching vibration peak of the structural hydroxyl group (-OH) in kaolin appeared at 3600–3700 cm^−1^, confirming that an appropriate amount of slag effectively stimulates the hydrolysis and polymerization of kaolin components in CG [30]. Combined with the data in Table 5, the 30% CG content group showed the highest compressive strength. This indicates that C-(A)-S-H gel was more effective in constructing early strength and a dense structure. Figure 9b shows that the modulus change had little effect on the Si-O-T peak position, and only a slightly higher wavenumber shift occurred when the modulus was high. This is consistent with the conclusion that the effect of the modulus on strength was less than that of the CG content in the range analysis, indicating that the effect of modulus on the chemical composition of the gel was relatively weak. Figure 9c shows that when the content was less than 8%, the band change was small, which is conducive to the formation of the Si-O-T structure; after more than 8%, the characteristic peak moved to the high wave number, indicating that excessive alkali activator will inhibit the hydrolysis of solid wastewater and interfere with the formation of gel. Upon further increasing the amount of sodium oxide, the strong alkaline environment promoted the conversion of high-polymerization-degree silicate to low-polymerization-degree silicate, promoting the formation of many Si-O-T networks, and the characteristic peaks were significantly shifted to low wavenumbers.

As shown in Figure 9, all samples had a Si-O-Si bending vibration peak at 471 cm^−1^ [31] and an O-H vibration peak of gel-bound water near 1619 cm^−1^ and 3450 cm^−1^ [32]. The weak C-O vibration peak at 1436 cm^−1^ was due to slight carbonization on the surface. It can be reasonably inferred that the carbonate content in the hydrolysis polymerization product was extremely low or almost absent, which further supports the conclusion of the XRD phase analysis. Therefore, the rational design of slag content, modulus, and content of alkali activator can promote the connection polymerization of silicon-aluminum-oxygen tetrahedra and realize the synergistic formation of geopolymer crystal phase and amorphous phase.

#### 3.3.3. SEM Results

**Figure 10:**
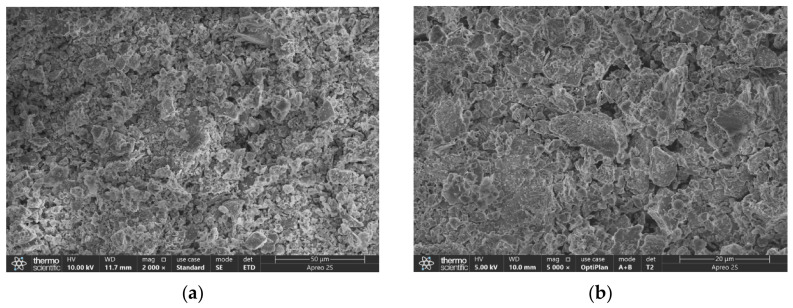

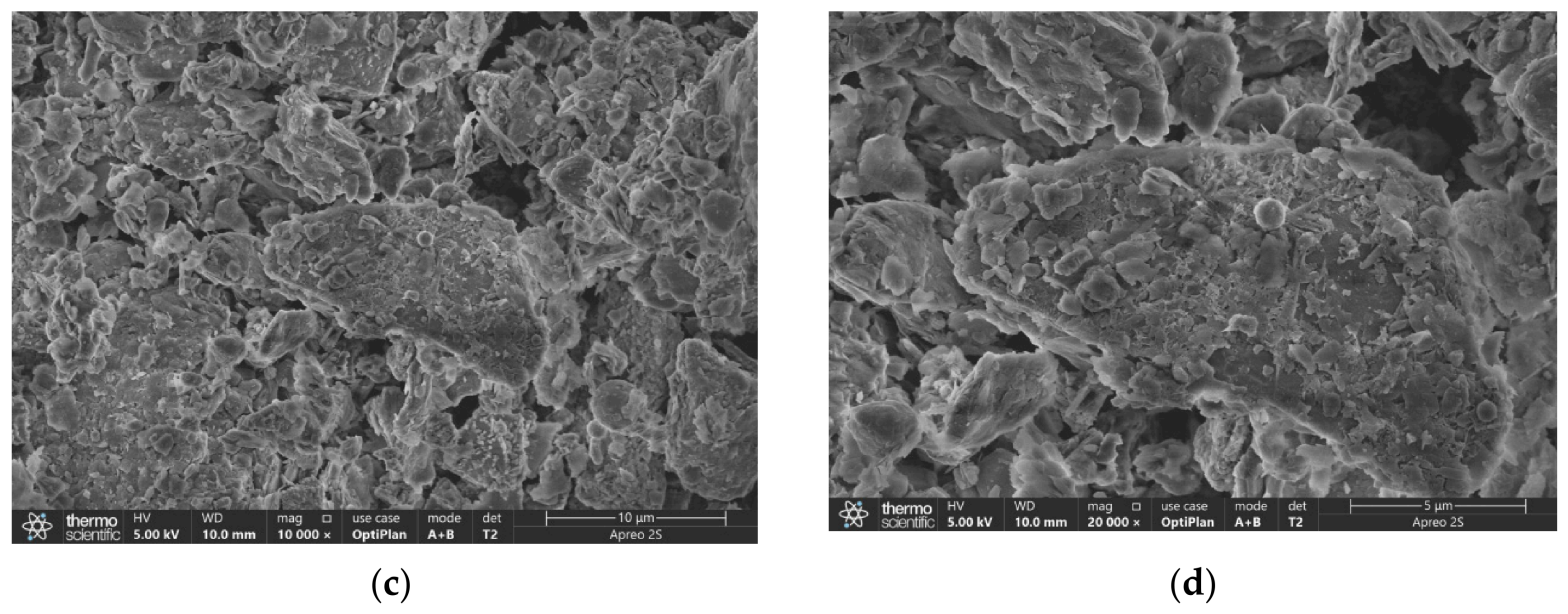
SEM image of AACGM with optimal mix ratio: (**a**) 2000, (**b**) 5000, (**c**) 10,000, and (**d**) 20,000 times.

**Figure 11:**
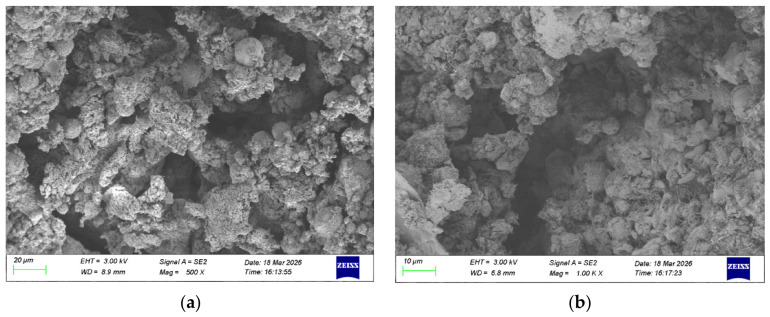
SEM image of sample 9: (**a**) 500, (**b**) 1000 times.

The hydrolysis process of CG-based geopolymer grouting material with an optimal mix ratio was systematically observed using SEM. Figure 10 and Figure 11 are the SEM images of the CG-based geopolymer grouting material with optimal mix ratio and number 9. The two show significant differences in microstructure, revealing the degree of hydrolysis-polymerization reaction and the internal law of gel structure evolution under different mix ratios.

As shown in Figure 10, from 2000 to 20,000 multi-scale microscopic observations, it was found that the microstructure of the material was dense after activation, and a large number of quartz phases were observed under high magnification, which was consistent with the XRD results. During the hydrolysis process, agglomerated, flocculent, and lamellar cementing products were formed in the outer layer. This indicates that the hydrolysis-polymerization reaction proceeds more thoroughly, resulting in the formation of a complex three-dimensional network gel structure. This phenomenon can be attributed to the hydrolysis of slag under the combined action of the alkali activator and NaOH, which generates a dense C-A-S-H gel that significantly contributes to the early strength development. CG and FA formed N-A-S-H gel by hydrolysis polymerization, released active components during long-term hydration, participated in the formation of C-(N)A-S-H gels, and promoted the development of later strength. The synergistic effect of the three bonded the four surfaces of [AlO_4_]^5−^ and [SiO_4_]^4−^ to each other to construct a continuous three-dimensional network gel structure. This ratio scheme fully demonstrates the comprehensive advantages of CG, FA, and slag materials.

In contrast, the microstructure of Sample No. 9, as shown in Figure 11, appears loose and disordered, with a significant number of unreacted coal gangue and fly ash particles. The surfaces of these particles remain smooth and exhibit no visible signs of erosion, while pores are clearly developed throughout the matrix. This indicates an ineffective alkali activation process, wherein the dissolution of reactive components is limited, leading to insufficient formation of C-A-S-H and N-A-S-H gels. As a result, a well-connected three-dimensional gel network capable of providing structural strength fails to develop. The resulting loose microstructure directly contributes to the material’s low compressive strength.

In summary, the stark contrast between Figure 10 and Figure 11 clearly demonstrates the decisive role of mix proportion design in determining the microstructure and macroscopic performance of geopolymer materials.

## 4. Conclusions

In this paper, an AACGM was prepared. The main conclusions of this study regarding the influence of different factors on performance are as follows:

(1) Through range analysis, it is concluded that the fluidity of geopolymer grouting material decreases with an increase in CG content, and the modulus of alkali activator and the content of alkali activator increase with an increase in CG content. The setting time increased with an increase in the CG content and alkali activator modulus, and the alkali activator content first increased and then decreased. The compressive strength decreased with increasing CG content and increased with increasing alkali activator modulus and alkali activator content. Impermeability decreased with an increase in CG content, alkali activator modulus, and alkali activator content.
(2) Further analysis of the range analysis by orthogonal matrix showed that the optimal mix ratio parameters of geopolymer grouting materials are a CG content of 50%, an alkali activator modulus of 1.6, and an alkali activator content of 14%. The order of influence of various factors on geopolymer grouting materials was as follows: CG content > alkali activator content > alkali activator modulus. Among them, CG content significantly affected the compressive strength and impermeability of the material. The alkali activator content mainly affected the setting time, and the modulus of the alkali activator had the greatest influence on fluidity.
(3) Through XRD, FTIR, and SEM analyses, it was found that the stone structure formed by AACGM is compact and its porosity is low. The main hydrolysis polymerization products included a zeolite-like phase, C-S-H gel, and C-(N)-A-S-H gel. The mechanical properties of the CG-based geopolymer were improved. In the early stage, the pore structure was optimized as an inert filler, and in the later stage, highly active hydration products were generated by chemical conversion. This provided a new idea for the utilization of industrial solid waste resources and was suitable for the development of grouting materials that should consider both strength and impermeability in underground engineering.

This study confirmed the technical feasibility of using CG in the preparation of grouting materials, determined the optimal ratio via orthogonal testing, and revealed the gel formation mechanism. In addition, a follow-up study will conduct a life cycle assessment of the high energy consumption of the multi-stage grinding process to comprehensively verify its environmental benefits. Machine learning was introduced to optimize the ratio [33]. Quantitative analysis using techniques such as X-ray full-spectrum fitting and solid-state nuclear magnetic resonance will be employed to investigate the gel evolution law and evaluate the long-term stability of the mineral phases. In parallel, a systematic study of the material’s rheological properties and injectability will be conducted, and durability tests under multi-factor coupling will be conducted to provide more comprehensive data support for underground engineering applications.

## Figures and Tables

**Table 1:** Material chemical composition/wt.%.

Chemical Composition	SiO_2_	Al_2_O_3_	Fe_2_O_3_	MgO	CaO	K_2_O	Na_2_O	SO_3_
CG	57.76	30.6	4.33	1.02	0.21	2.77	1.1	0.17
FA	60.12	22.07	5.31	1.61	6.17	1.63	0.14	1.05
Slag	33.66	16.81	1.14	6.51	37.65	0.59	0.71	1.44

**Table 2:** Orthogonal test settings.

Sample Number	Factor A CG Admixture Ratio	Factor B Alkali Activator Modulus	Factor C Alkali Activator Dosage
1	30%	1.2	12%
2	50%	1.6	14%
3	70%	2	16%

**Table 3:** Trial mix proportions.

Sample Number	CG Admixture Ratio (kg·m^−3^)	Alkali Activator Modulus	Alkali Activator Dosage (%)	Slag Admixture Ratio (kg·m^−3^)	FA Admixture Ratio (kg·m^−3^)
1	180	1.2	12	360	60
2	180	1.6	16	360	60
3	180	2	14	360	60
4	300	1.2	16	240	60
5	300	1.6	14	240	60
6	300	2	12	240	60
7	420	1.2	14	120	60
8	420	1.6	12	120	60
9	420	2	16	120	60

**Table 4:** Mix proportion design and test results of geopolymer grouting material.

Sample Number	A (%)	B	C (%)	Flow (mm)	Compressive Strength (MPa)	Setting Time (min)	Seepage Pressure (MPa)
1	30	1.2	12	190	40.43	190	1.9
2	30	1.6	16	252	41.87	298	1.6
3	30	2	14	246	40.23	448	1.8
4	50	1.2	16	218	37.46	433	1.7
5	50	1.6	14	216	37.51	548	1.6
6	50	2	12	212	35.63	483	1.4
7	70	1.2	14	171	35.37	458	1.3
8	70	1.6	12	192	32.56	554	1.6
9	70	2	16	224	29.76	474	1.2

**Table 5:** Range analysis results.

Test Method	Factor	r1	r2	r3	R	Primary and Secondary Factors	Excellent Scheme
Flow	A	229.33	215.33	195.67	33.66	B > A > C	A_1_B_3_C_3_
	B	193	220	227.33	34.33		
	C	198	211	231.33	33.33		
Setting Time	A	312	488	504.33	192.33	A > C > B	A_1_B_1_C_3_
	B	360.33	466.67	468.33	108		
	C	409	516.67	401.67	115		
Compressive Strength	A	40.84	36.87	32.57	8.27	A > B > C	A_1_B_1_C_2_
	B	37.75	37.31	35.21	2.54		
	C	36.21	36.77	36.37	0.56		
Seepage Pressure	A	1.77	1.57	1.37	0.4	A > B > C	A_1_B_1_C_1_
	B	1.63	1.6	1.47	0.16		
	C	1.63	1.57	1.5	0.13		

**Table 6:** Optimal mix ratio performance parameters.

Sample Number	Flow (mm)	Setting Time (min)	Compressive Strength (MPa)	Seepage Pressure (MPa)
ZJ	246	313	36.23	1.8

## Data Availability

The original contributions of this study are included in this article. Further inquiries can be directed to the corresponding author.

## Abbreviations

The following abbreviations are used in this manuscript:

AACGM	Alkali-Activated Coal Gangue-Based Geopolymer Grouting Material
CG	Coal Gangue
FA	Fly Ash
XRF	X-Ray Fluorescence
XRD	X-Ray Diffraction
FTIR	Fourier Transform Infrared Spectroscopy
SEM	Scanning Electron Microscope
